# Digesting an ancient ecosystem: coprolites from the Grippia bonebed, Lower Triassic, Svalbard

**DOI:** 10.7717/peerj.20746

**Published:** 2026-02-17

**Authors:** Vanja Simonsen, Aubrey Jane Roberts, Victoria Sjøholt Engelschiøn, Øyvind Hammer, Jørn H. Hurum

**Affiliations:** 1 Natural History Museum, University of Oslo, Oslo, Norway; 2 Fossil Reptiles, Amphibians and Birds Section, The Natural History Museum, London, United Kingdom

**Keywords:** Coprolites, Svalbard, Triassic, Paleoecology, CT, Vikinghøgda Formation

## Abstract

The marine Grippia bonebed from Vikinghøgda Formation, Lower Triassic, Svalbard is composed of scattered skeletal remains, teeth and coprolites. From this, five coprolite morphotypes have been identified and described. In this study 97 coprolites were examined and classified based on their morphology and inclusions. Thin sections, scanning electron microscope (SEM) and micro-computed tomography (micro-CT) were used to analyze the inclusions revealing skeletal material, fish scales and notably the first observations of invertebrates in this locality. Among the invertebrate components were sponge spicules, a fragment of a cephalopod shell and numerous onychites. Potential coprolite producers are evaluated and likely include chondrichthyans, osteichthyes, ichthyopterygians and temnospondyls. Additionally, this research provides a CT-scanning method for identifying low-density inclusions such as onychites and contribute to a better understanding of the marine Early Triassic paleoecosystem and food web on Svalbard.

## Introduction

Coprolites (fossilized feces) are valuable for paleoecosystem reconstruction and provide information on ecological interactions, animal physiology and behavior (Niedźwiedzki et al., 2016b; Bajdek & Bienkowska-Wasiluk, 2020; Mingtao et al., 2022) as well as parasite-host associations and coprophagy (Milàn, 2012). Early Triassic coprolites are rare and have previously been found only in a few localities in Australia, South Africa and Europe (Hunt, Lucas & Spielmann, 2013). Northwood (2005) studied Early Triassic coprolites from Australia containing rarely preserved organisms such as cyanobacteria and arthropods in addition to the more commonly preserved fish, highlighting the unique preservation potential of coprolites. Invertebrate remains, such as bivalves, have also been found in Triassic coprolites from South Africa (Yates, Neumann & Hancox, 2012). Coprolites can better our understanding of the environment and paleoecosystems after the Permian-Triassic extinction (Niedźwiedzki et al., 2016a). For example coprolites from Poland filles gaps in the fossil record, suggesting that marine predation started very early in the Mesozoic Marine Revolution (Brachaniec et al., 2015). Research on Early Triassic coprolites show a variety of morphotypes such as rounded, cylindrical, spirals and striated, with the most common producers being Osteichthyes, chondrichthyans, temnospondyls and archosauromorhs (Northwood, 2005; Yates, Neumann & Hancox, 2012; Brachaniec et al., 2015; Niedźwiedzki et al., 2016a).

This study examines coprolites collected from the Grippia bonebed material in Svalbard, aiming to uncover new information about the bonebed’s paleoecosystem after the Permian-Triassic mass extinction. Possible producers of the coprolites will be discussed based on morphotypes and inclusions, although coprolites are difficult to link to their producer; it is usually possible to assume identification at higher taxonomic levels (Hunt, Lucas & Spielmann, 2013; Brachaniec et al., 2022).

### Geological setting

The Grippia bonebed is a fossiliferous horizon in the lower Vendomdalen Member deposited in an Early Triassic marine environment. Svalbard is today located at about 78–84 degrees north but was in the Early Triassic at about 45 degrees paleolatitude in the Boreal Ocean of northern Pangea (Hurum et al., 2018). The Vikinghøgda Formation consists of the Deltadalen and Lusitaniadalen members (Induan) and the Vendomdalen Member (Olenekian), and was deposited in a shallow marine shelf environment that was getting deeper over time (Hansen, Hammer & Nakrem, 2018).

The Grippia Niveau was first described by Stensiö (1921) and later named by Wiman (1928) after the basal ichthyosaur *Grippia longirostris.* The locality excavated in Flowerdalen can not be exactly correlated to the original Grippia Niveau described by Stensiö, but we use the terminology as our bonebed has abundant *Grippia* remains. Our Grippia bonebed has a thickness of five cm or less and consists of unconsolidated, phosphate rich material with disarticulated vertebrate bones, teeth, and coprolites deposited in a period of low oxygen conditions (Hansen, Hammer & Nakrem, 2018). The bonebed is of early Spathian age as supported by conodont element analysis (Nakrem & Orchard, 2023).

**Figure 1:**
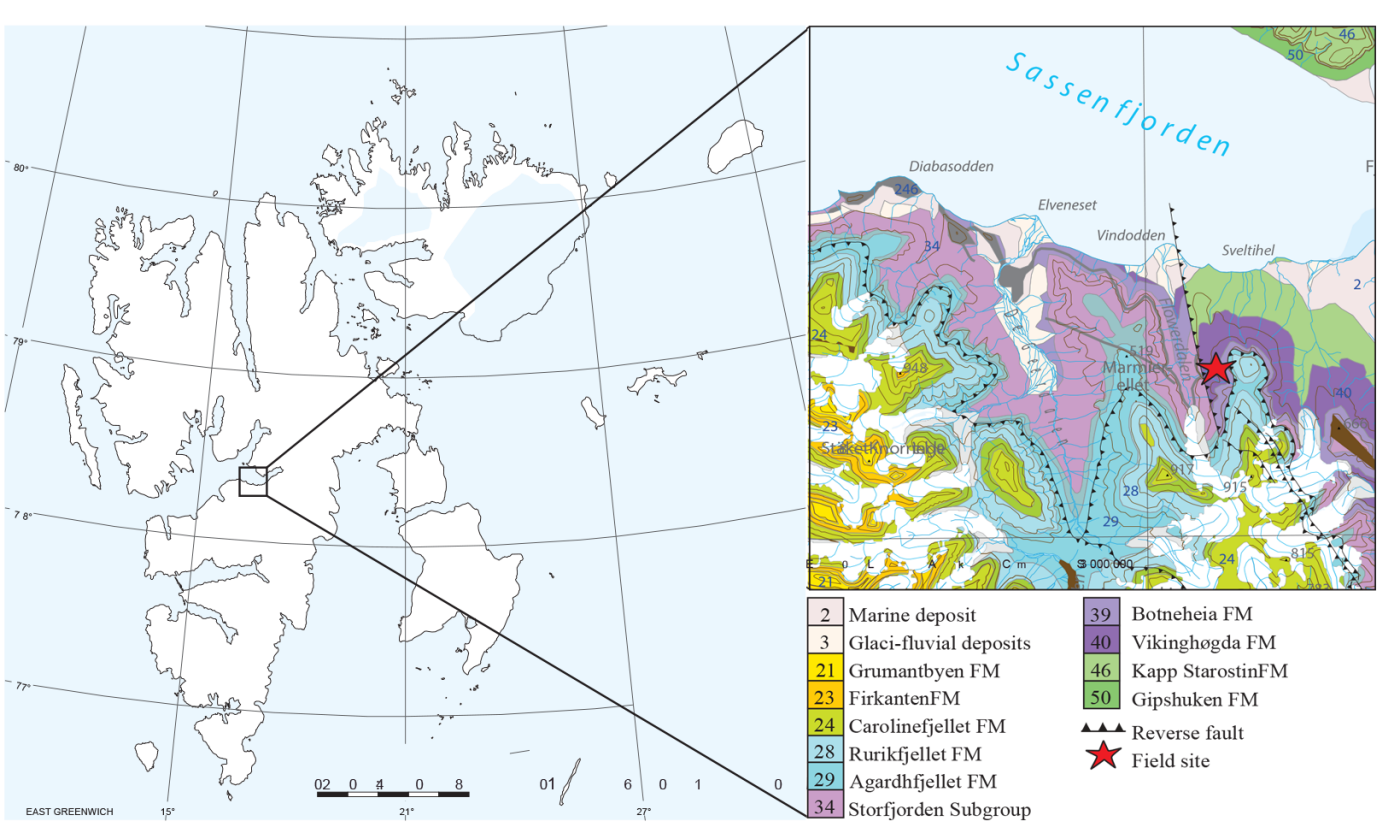
Map of the study area, Grippia bonebed, Svalbard.

The bonebed was excavated at Marmierfjellet near Flowerdalen, central Spitsbergen in 2015 and 2016 by the Spitsbergen Mesozoic Research Group from the Natural History Museum of Oslo (Fig. 1). For more detailed stratigraphy and discussions on depositional environment see Hansen, Hammer & Nakrem (2018) and Nakrem & Orchard (2023).

## Materials & Methods

250–300 kg of material was recovered from the Grippia bonebed by The Spitsbergen Mesozoic Research Group in 2015 and 600 kg in 2016 at Marmierfjellet (Bratvold, Delsett & Hurum, 2018; Ekeheien et al., 2018). All necessary excavation permits were obtained from the Governor of Svalbard permit no. 2013/01222-2. The material is stored at the Natural History Museum of Oslo, Norway. The material was sieved and sorted into skeletal remains, teeth, and coprolites. Most of the coprolite material has been crushed during re-deposition or fragmented by congelifraction and collection, as indicated by fresh cleavages. About half of the coprolite material was investigated for complete or almost complete specimens. From the 1000s of coprolite fragments, 97 coprolites are included in this study, and 5 broken specimens were kept as examples of typical broken pieces. When selecting coprolites, the focus was on specimens that best represent each morphotype according to their typical shapes, and variety of shapes and terminations. Coprolites were chosen to also show the size range, but the number of coprolites of each size is not representative. Size and shape were not investigated statistically because of the extensive breakage and the highly biased sampling. The morphotypes were described using a combination of shape, surface texture and inclusions. Morphotype descriptions and terminology in this paper are partly based on Hunt & Lucas (2012). The most representative specimens were photographed using photo stacking with a Nikon D850 with Nikon AF-S micro nikkor 60 mm 1:2.8 lens and processed in Adobe Photoshop version 2024.

### Micro CT-scanning

Twenty-three CT-scans of different morphotypes were made using the Nikon Metrology XT H 225 ST instrument at NHM, Oslo and processed in Avizo 2022. Scans were studied using orthoslice and 3D-models in both normal and inverted view. Parameters for each scan are noted in supplementary (Table S1).

Due to CT-pictures being in 3D a scale is only used when focusing on a specific inclusion, otherwise the scale would be inaccurate, but sizes of specific fragments are stated in the figure text.

### Transmitted light microscopy

Specimens for petrological thin sections were embedded in epoxy. Only half the specimen was used for making thin sections, with the other half kept in the collections for future studies. Sixteen thin sections were studied and photographed using a Leica DMPL microscope with Leica camera MC170HD and processed in Leica Application Suite (LAS). No thin sections were made of morphotypes A3 and B1 due to the low sample size and as their unique, characteristic features that would have been lost in the process.

### Chemical analyses

Chemical analyses were done with a Hitachi 300600N Scanning Electron Microscope (SEM) equipped with Energy Dispersive Spectrometer (EDS), using point analyses and chemical mapping on three specimens. The specimens were chosen to represent a variety of coprolite inclusions and morphotypes.

Information about which specimens were CT-scanned, thin sectioned and photographed is summarized in supplementary (Table S2).

## Results

**Figure 2:**
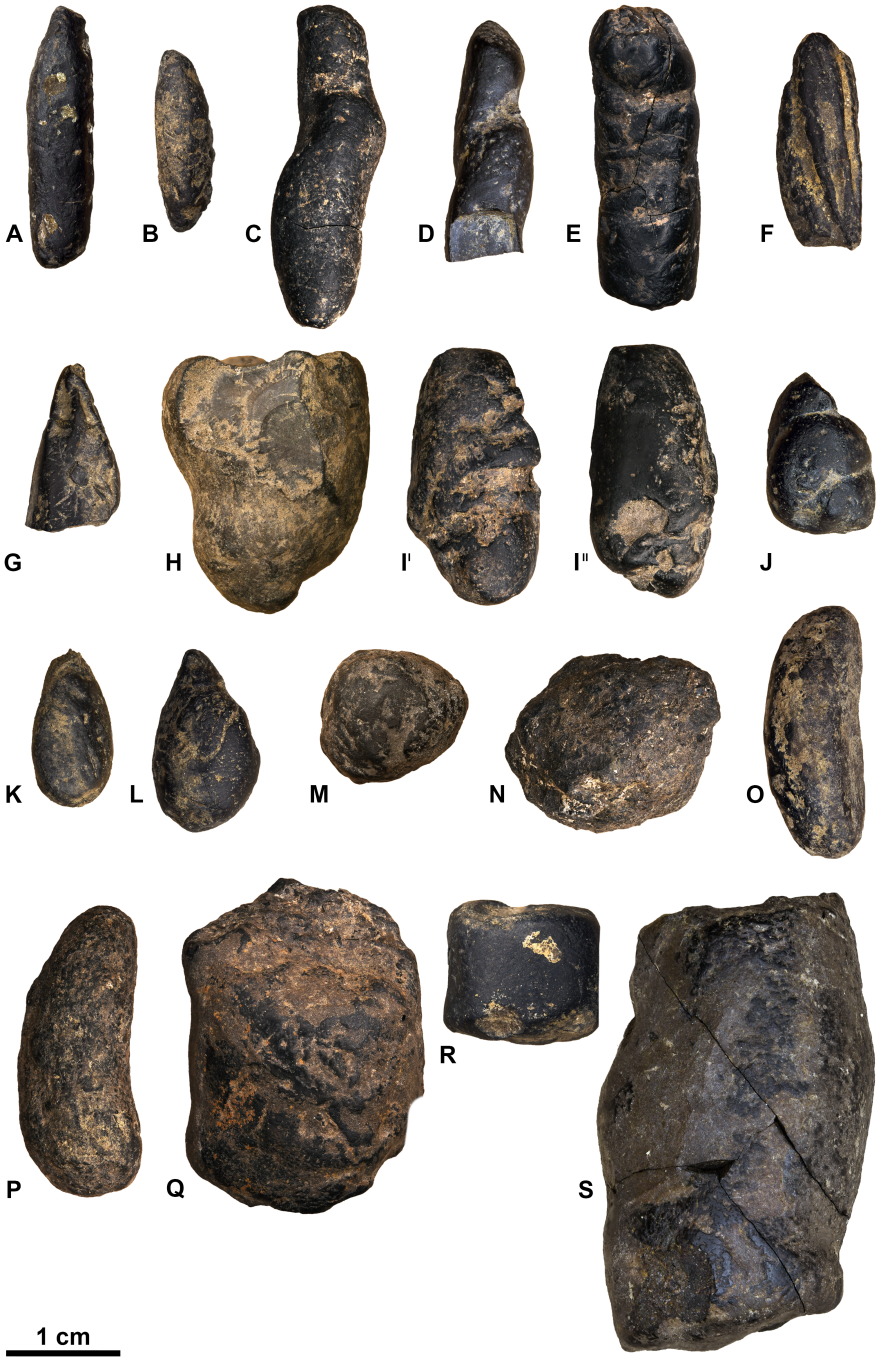
Photographs of the different morphotypes found in the Grippia bonebed, Svalbard. (A–B) morphotype A1 cigar. (C–E) morphotype A2 amphipolar. (F–G) morphotype A3 cylindrical with grooves. (H–J) morphotype B1 spiral rounded. (K–L) morphotype B2 teardrop. (M–N) morphotype C sub-rounded. (O-P) morphotype D reniform. (Q–S) morphotype E wide cylindrical.(A) PMO 250.847. (B) PMO 250.846. (C) PMO 250.275. (D) PMO 250.854. (E) PMO 250.858. (F) PMO 250.860. (G) PMO 250.530. (H) PMO 250.533. (I’-I”) PMO 250.864. (J) PMO 250.841. (K) PMO 250.869. (L) PMO 250.868. (M) PMO 250.271. (N) PMO 250.884. (O) PMO 250.886. (P) PMO 250.528. (Q) PMO 250.899. (R) PMO 250.897. (S) PMO 250.281.

Ninety-six coprolites were sorted into five morphotypes (Morphotype A-E, Fig. 2) with a total of eight sub-types summarized in Table 1. An additional uncategorized specimen (PMO 250.282) is included in this study because of its unique inclusions. The coprolites frequently exhibit fish scales adhered to the surface. Due to the regular appearance of fish scales in the bonebed matrix as well as on the surface of skeletal material, these have not been classified as inclusions and likely originate from the surrounding matrix.

**Table 1:** Abundance of inclusions by morphotype. Numbers indicate the number of specimens.

Morph.type	Sub-morphotype	Illustration	Length in mm	Description	Inclusions	Producers	Total N
A	A1 Cigar	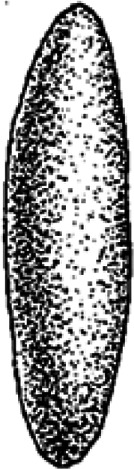	11–35	Elongated, cylindrical, straight, anisopolar (sometimes isopolar)	Fish scales, fish bones ex. Vertebrae, tetrapod bone?	Fish	15
	A2 Amphipolar	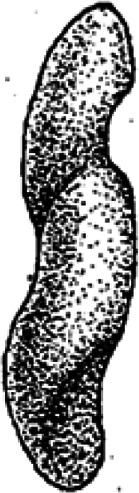	11–34	Elongated, cylindrical with amphipolar spiral or twist, anisopolar and isopolar	Fish scales, fish bones ex. Vertebrae and neaural arch, onychites	Fish, ex. Dipnoi, coelacanths, sauricthyes	14
	A3 Cylindrical, grooves	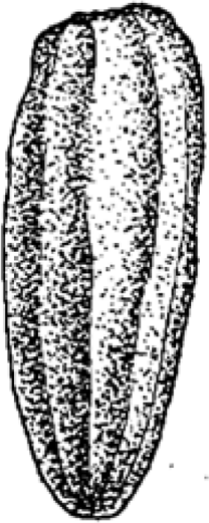	17–95	Elongated, cylindrical with grooves, anisopolar, slowly tapering	Bone fragments, conodont elements, shark tooth	Archosauromorphs	6
B	B1 Spiral, rounded	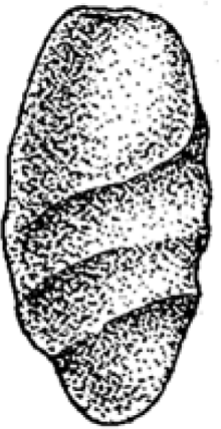	10–29	Oval to drop shaped, spiral, anisopolar with rounded and slightly tapered ends	Fish scales, bone fragments, ichtyopterygian tooth?	Sharks	8
	B2 Teardrop	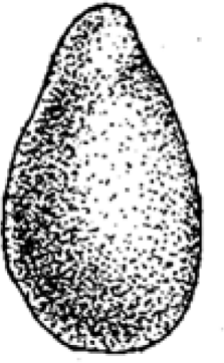	7–20	Wide at one end and tapered on the other end	Fish scales, fish bones?, onychites, sponge spicules	Fish?	14
C	C Sub-Rounded	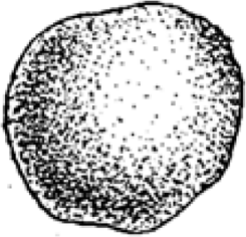	7–40	Sub-spherical, slightly flattened	Fish bones? Tetrapod bone?	Fish? Temnospondyls?	15
D	D Reniform	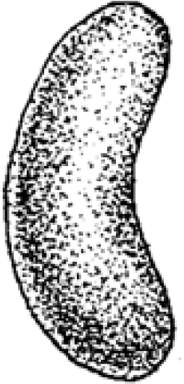	13–29	Bean shaped, rounded ends, isopolar	Fish scales, bone fragments, onychites	Reptile? Temnospondyls?	10
E	E Wide cylindrical	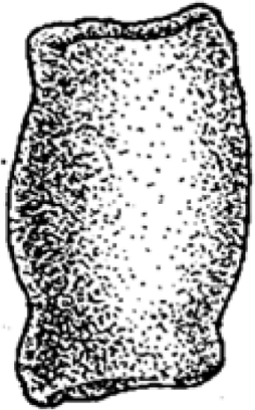	12–40	Cylindrical, isopolar with flat or concave ends	Bone fragments, onychites and orthoceratoid shell	Ichthyopterygians?	13

### Morphotype A: elongated cylindrical

#### Morphology

**Figure 3:**
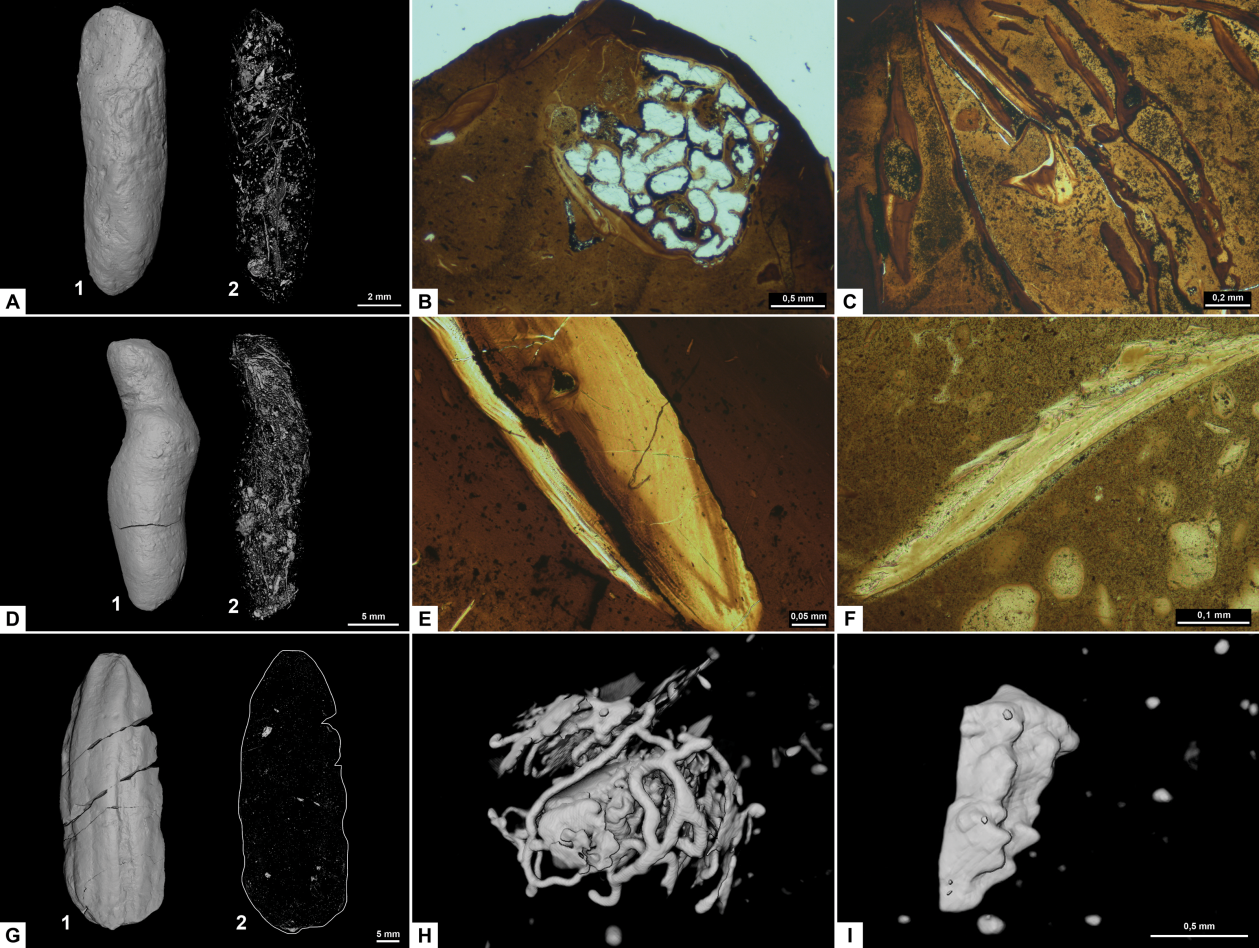
CT-scans (A, D, G–I) and thin sections (B–C, E–F) of morphotype A. (A1–A2) External and internal view of sub-morphotype A, PMO 250.270. (B) Tetrapod bone in thin section, PMO 250.004. (C) Fish vertebrae, PMO 250.009. (D1–D2) External and internal view of sub-morphotype A2, PMO 150.275. (E) Fish scale, PMO 249.999. (F) Degraded bone fragments, PMO 250.000. (G1–G2) External and internal view of sub-morphotype A3, PMO 250.273. (H) Burrows with a width of approximately 0.5 mm over a total area of three mm, PMO 250.530. (I) Conodont element measuring 1.22 mm in length, PMO 250.273.

Morphotype A (Fig. 3) is elongated cylindrical with a near-constant diameter and typically displays an anisopolar configuration, with one end being rounded and the other slightly tapered. The morphotype is divided into three sub-types: A1 cigar (Fig. 3A), A2 amphipolar, where the whole length of the specimen shows an even spiral (Fig. 3D) (Hunt & Lucas, 2012); A3 cylindrical with grooves (Fig. 3G).

Variants A1 and A2 may occasionally be isopolar, possessing either two rounded or two tapering terminations. Sub-morphotypes A1 and A2 are rarely flattened and usually have a polished black surface. The overall shape of A1 is akin to a straight cigar with a smooth surface (Fig. 3A). In contrast, A2 is characterized by an amphipolar spiral that coils 1–2 times (Fig. 3D). Some A2 specimens such as PMO 250.858 (Fig. 2E), have an uneven structure on the surface interpreted to be tight spiraling. This uneven pattern indicates that this sub-morphotype might have either a tight or loose spiral configuration. Spiral structures should be visible in thin sections (Jain, 1983), but this was not found. Morphotype A3 is rare and distinguished by longitudinal grooves that may run partially or entirely along the coprolite (Fig. 3G) as typical constriction marks (Northwood, 2005), and specimens are usually flattened. Specimen PMO 250.267 (Fig. S1) is the largest specimen in this study, measuring 95 mm in length and 45 mm in width, and fits the description of morphotype A3 with distinct grooves.

#### Inclusions

Inclusions for sub-morphotypes A1 are described by two CT-scans and five thin sections; A2 by three CT-scans and two thin sections; A3 by four CT-scans. The inclusions in all studied coprolites are evenly distributed with the largest fragments generally located toward the edges of the coprolite. The number of inclusions varies significantly within sub-morphotypes A1 and A2, with some specimens being almost empty while others are filled with angular inclusions both in CT and thin sections. The inclusions have a low grade of degradation. Sub-morphotype A3 is for the most part homogenous except for a few small fragments (Fig. 3G).

Most inclusions in CT were unrecognizable with a few exceptions in morphotypes A2 and A3 identified as fish bones, belemnoid hooks (onychites) (Hoffmann, Weinkauf & Fuchs, 2017), conodont elements, a possible shark tooth and a neural spine (Fig. 3, Fig. S2). A3 specimen PMO 250.530 is empty in CT-scan except for a collection of trace fossils in the middle of the specimen (Fig. 3H). The traces cover a total area of 3x3 mm and are typically around 0.5 mm wide. There are no signs of an entry point.

Seen in thin sections, sub-morphotypes A1 and A2 have many inclusions of bone fragments and fish scales in a fine-grained matrix. The fish scales are well preserved and have visible bone structure, growth lines, pores and enamel (Fig. 3E) (as described in Mingtao et al., 2022). The cross sections of fish scales are often cut in a way that shows the ridges on the scales surface (Fig. S2D). Bone fragments in thin sections are typically smaller and appear better digested than the fish scales in all coprolites (Fig. 3F). Many of the bone fragments found in the coprolites likely originate from fish because of their small size, and some have a strong resemblance to fish vertebrae (as seen in Mingtao et al., 2022) as well as having a high abundance in coprolites together with fish scales. Thin section of PMO 250.009 (A1) is packed with fish vertebrae similar to fig. 8C from Mingtao et al. (2022) as well as scales (Fig. 3C). PMO 250.004 (A1) contains two pieces of highly porous bone fragments typical for tetrapod bones (Fig. 3B). Bone structures like this suggests that the bone may be ichthyopterygia (Sander, 2021).

### Morphotype B: rounded spiral and teardrop

#### Morphology

**Figure 4:**
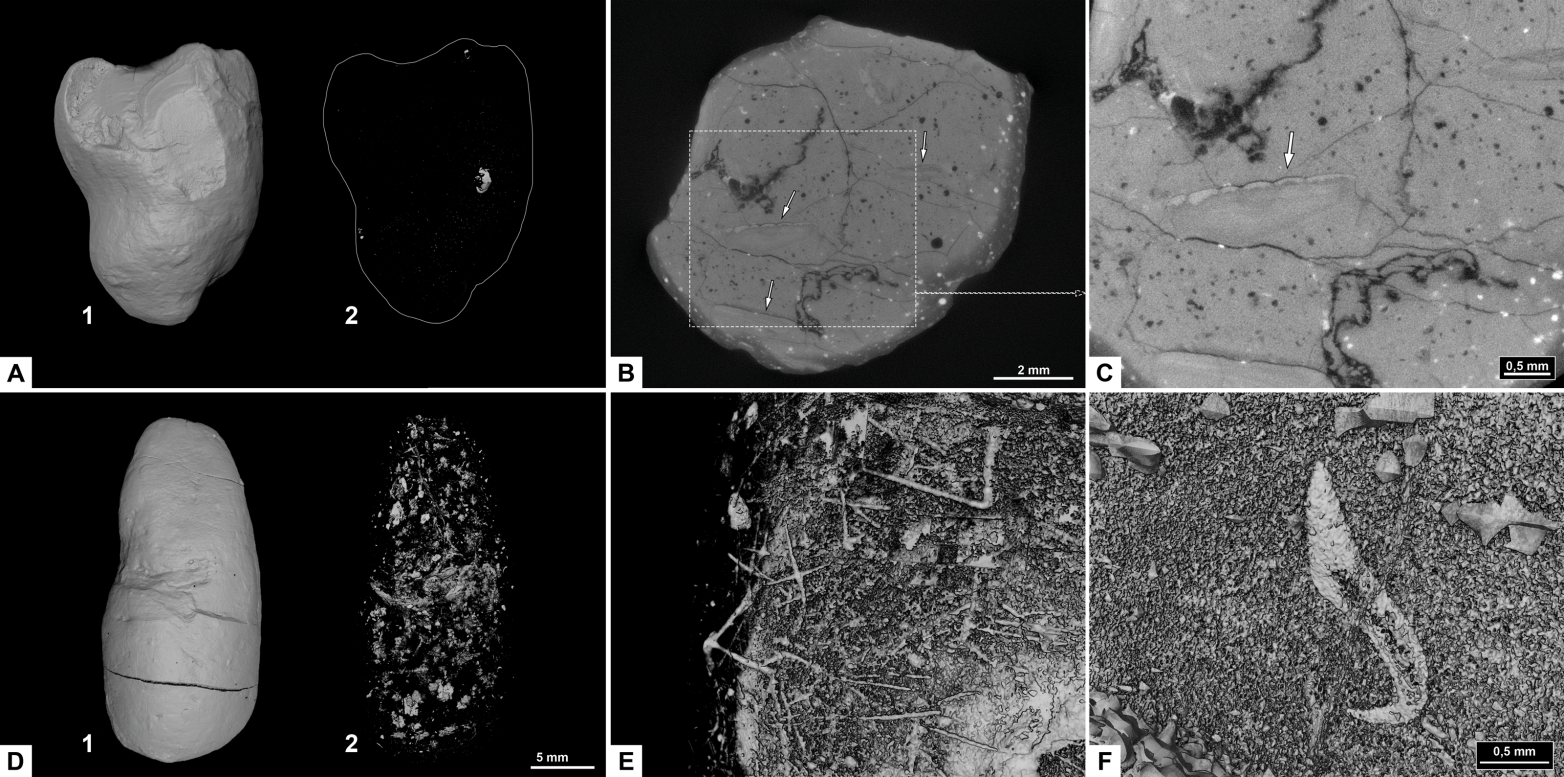
CT-scans of morphotype B. (A1–A2) External and internal view of sub-morphotype B1, PMO 250.533. (B) Orthoslice with white arrows pointing to fish scales, PMO 250.241. (C) Orthoslice with arrow pointing to fish scale with ridges, PMO 250.241. (D) External and internal view of sub-morphotype B2, PMO 250.265. (E) Sponge spicules measuring 0.5–1.0 mm, PMO 250.265. (F) Inverted CT-reconstruction of onychite measuring 2.3 mm in length, PMO 250.265.

Morphotype B (Fig. 4) is anisopolar, generally exhibiting a teardrop-like shape with one end broader than the other. The widest end may be rounded, flat or slightly concave. While a few specimens are somewhat flattened, the majority retain their rounded cross section.

Morphotype B is divided into B1 rounded spiral (Fig. 4A) and B2 teardrop (Fig. 4D). B1 is characterized by a heteropolar spiral which means that the spiral covers a maximum 75% of the coprolite at the posterior end (Hunt & Lucas, 2012). Four specimens might exhibit a more amphipolar condition but are poorly preserved. B1 usually has an overall teardrop shape with one exception that has an oval shape (PMO 250.865). Only PMO 250.841 (Fig. 2J) displays distinct spiral lines indicative of an amphipolar spiral. Six of the B1 specimens are either broken or end abruptly with a flat edge, obscuring spiral type determination. Sub-morphotype B2 shares B1’s overall shape, but differs with a smooth, polished surface that lacks any texture.

#### Inclusions

Inclusions in sub-morphotype B1 are described by three CT-scans, and B2 by four CT-scans and one thin section. Morphotype B includes fish scales and bone fragments. The inclusions are either angular or rounded. The bone fragments are likely from fish because of their co-occurrence with fish scales in the same specimens. Fish scales are usually more complete compared to bone inclusions which are highly degraded and fragmented.

Sub-morphotype B1 is almost empty with the exception of a couple of small, rounded fragments and fish scales (Figs. 4B, 4C) in CT scans. In contrast, morphotype B2 is packed with both rounded and angular inclusions in CT and thin sections. Two specimens in sub-morphotype B2 contain onychites in both the normal and inverted CT-reconstruction (Fig. 4F). PMO 250.265 is unique among all coprolites described in this study as it contains sponge spicules in CT (Fig. 4E). The spicules measure 0.5–1 mm and are straight or hexagonal.

### Morphotype C: sub-rounded

#### Morphology

**Figure 5:**
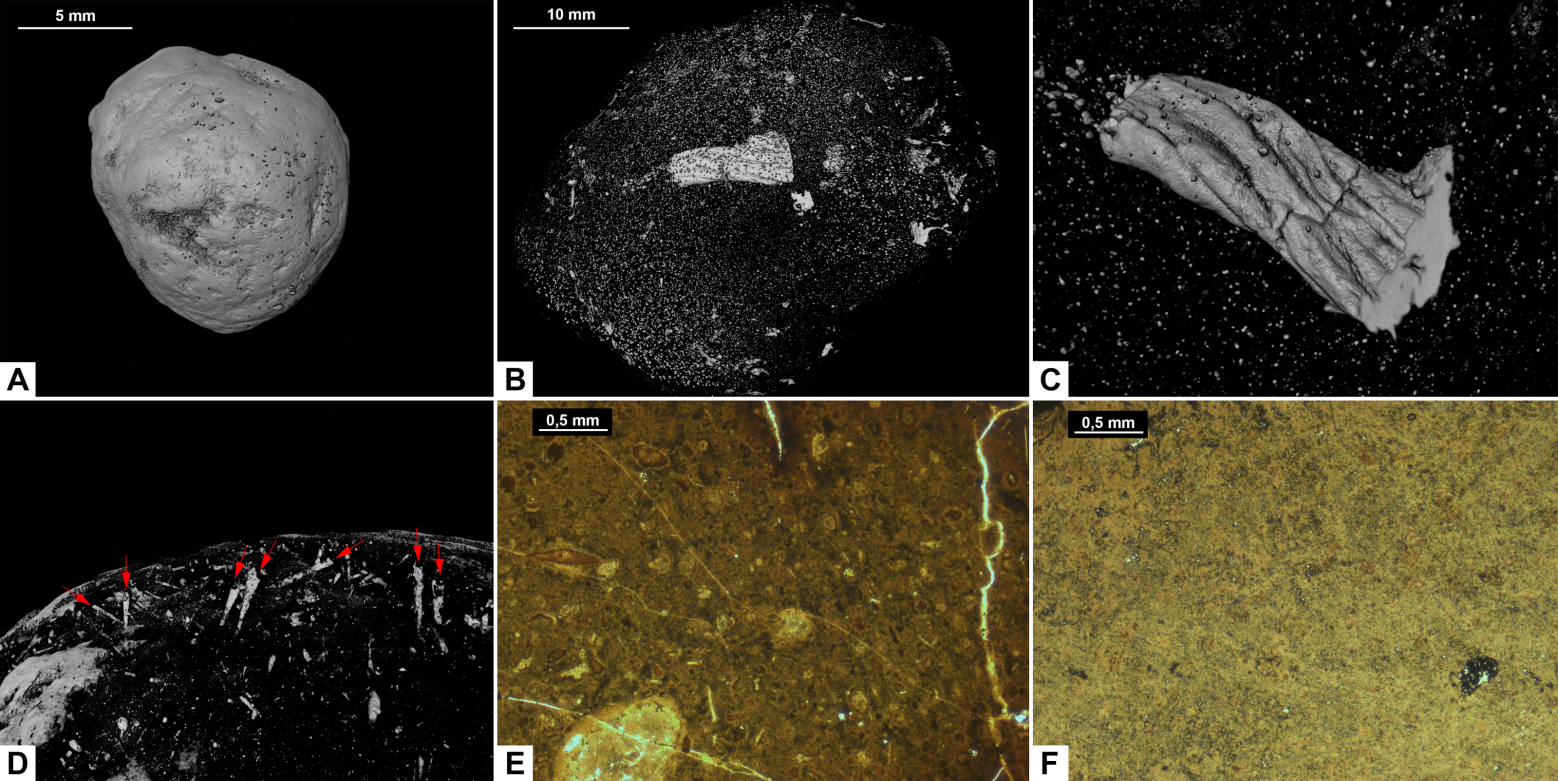
CT-scans (A–D) and thin sections (E–F) of morphotype C. (A) External view of morphotype C, PMO 250.271. (B) Internal view of PMO 250.277. (C) Unidentified fragment with length 10.7 mm, PMO 250.277. (D) Red arrows pointing to the entrance of cone shaped borings, PMO 250.0271. (E) Coarse matrix with degraded bone fragments, PMO 250.002. (F) Fine matrix with no inclusions, PMO 250.018.

The overall shape of morphotype C (Fig. 5) is sub-rounded, including specimens that are almost perfectly spherical (Fig. 5A) as well as slightly flattened specimens that appear oval. The sub-rounded coprolites have a perfectly smooth to irregular surface texture with the spherical specimens being the smoothest.

#### Inclusions

Inclusions in morphotype C are described by three CT-scans and four thin sections. Morphotype C is mostly homogenous in both CT-scans and thin sections with only a few rounded bone inclusions. The CT-scan of PMO 250.277 includes a few small fragments as well as one larger unidentified bone fragment measuring 10.7 mm in length (Fig. 5B). This large bone fragment has deep longitudinal grooves that gradually twist along its length. It seems abruptly broken off at one end and slightly fragmented on the other side (Fig. 5C). PMO 250.271 has a large number of cone-shaped borings inwards from the surface (Fig. 5D) in addition to a few small unidentified fragments. The widest part of the borings is located at the outer edges of the coprolite measuring 0.03 mm–0.16 mm in width and 0.8 mm–1.3 mm in depth. The only fragments seen in thin sections are small and degraded. Only PMO 250.002 has a coarse-grained matrix consisting of highly degraded bone fragments (Fig. 5E), while the remaining thin section specimens have a fine-grained matrix with no recognizable elements (Fig. 5F).

### Morphotype D: reniform

#### Morphology

**Figure 6:**
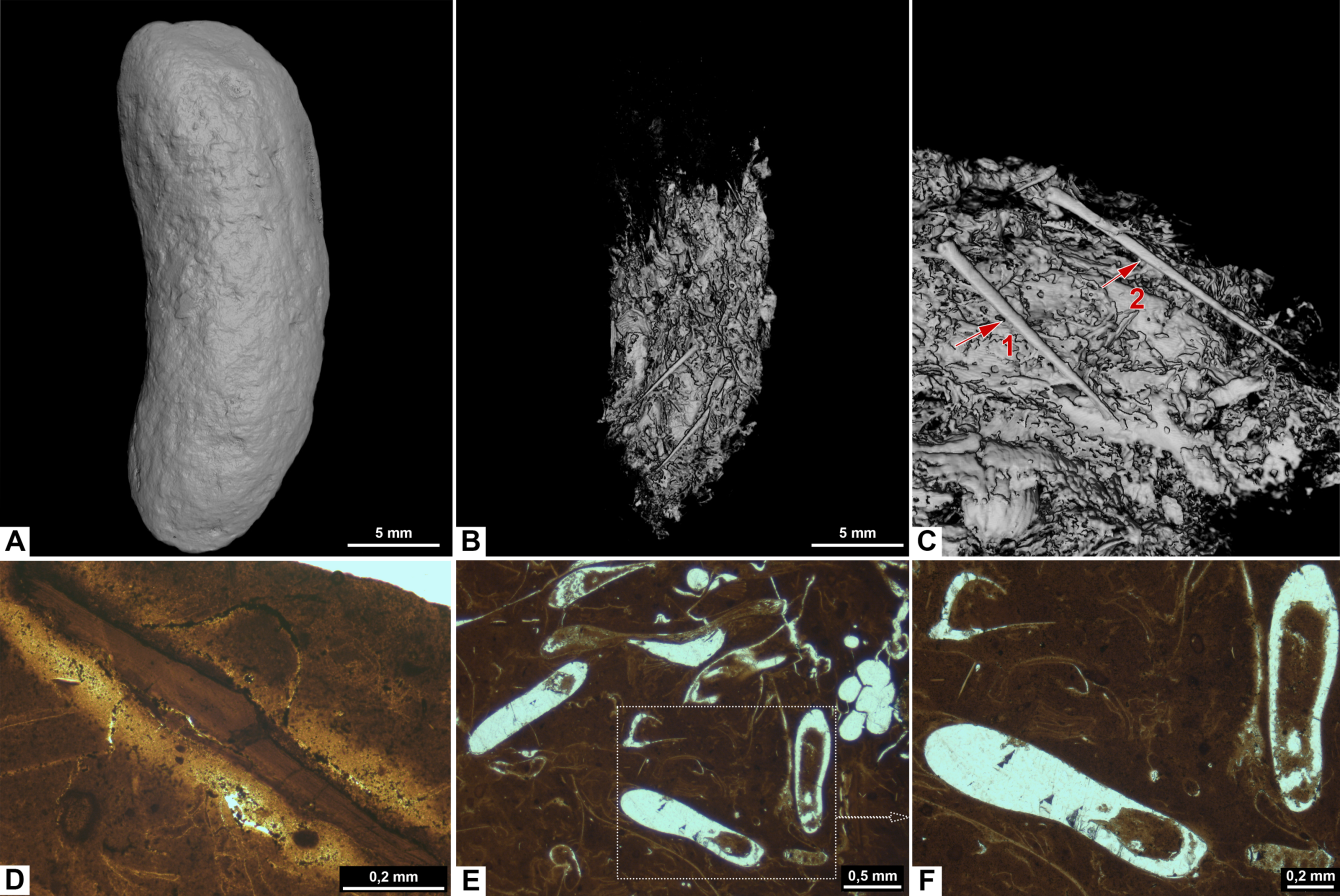
CT-scans (A–C) and thin sections (D–F) of morphotype D. (A) External view of morphotype D, PMO 250.528. (B–C) Internal view of PMO 250.528 Red arrows pointing to bone inclusions with arrow 1 measuring 3.6 mm and arrow 2 measuring 5.4 mm. (D) Bone fragment, PMO 250.005. (E–F) Mineralized inclusions and possible onychites, PMO 250.005.

Morphotype D (Fig. 6) has an overall reniform, or beanlike shape (Fig. 6A). They are isopolar with two rounded ends and typically have a smooth surface. Half of the specimens are flattened.

#### Inclusions

**Figure 7:**
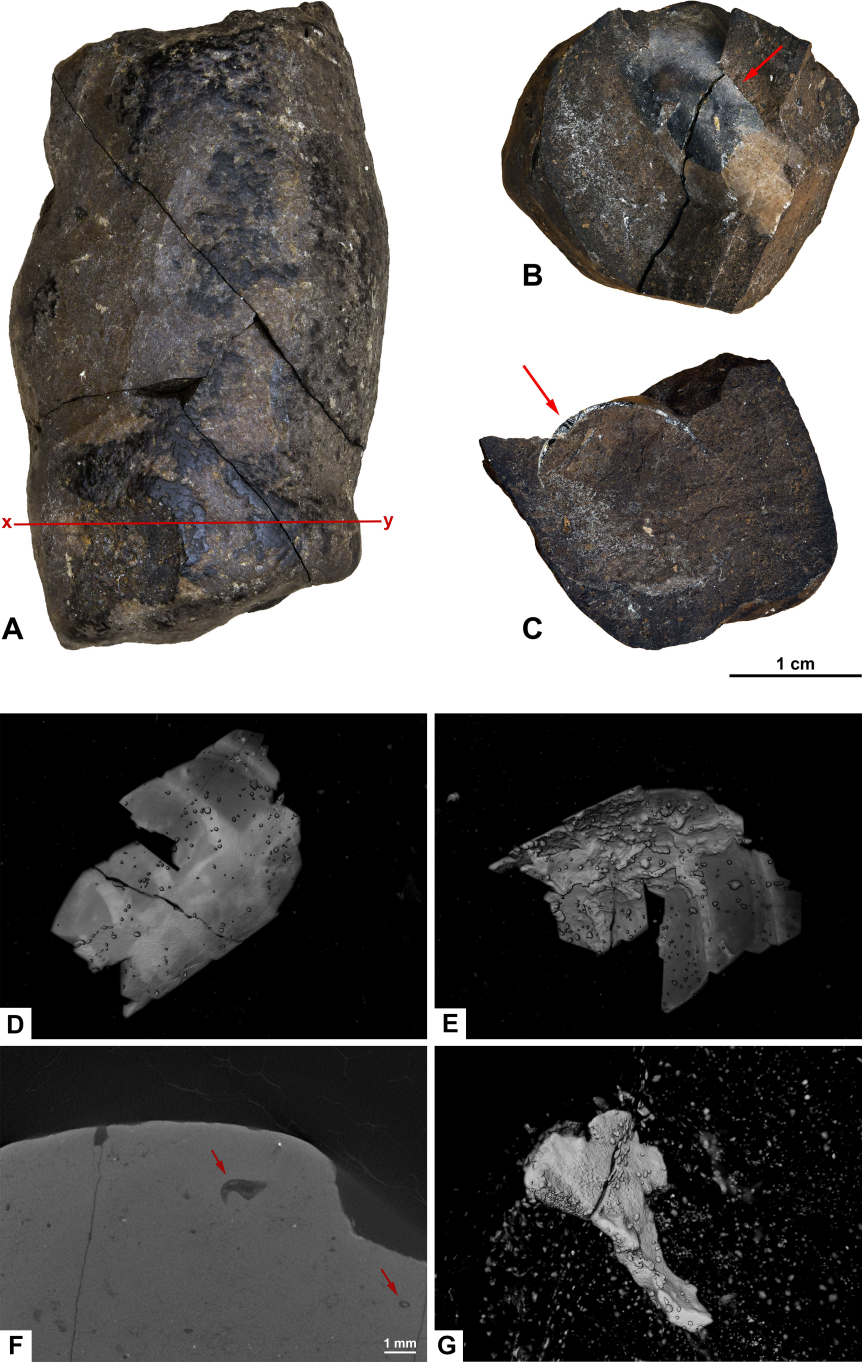
Photographs (A–C) and CT-scans (D–G) of morphotype E. (A) The complete specimen with *x-y* being cross section in picture F. (B–C) Orthoceratoid cephalopod shell fragment seen in two different angles. Red arrows pointing to the same spot in both pictures. (B) Shell fragment seen from the top. (C) Shell fragment seen in cross section. (D–E) Orthoceratoid shell fragment with a length of 15.8 mm and visible chambers. (D) Outside of the shell fragment. (E) Underside of the shell fragment. (F) Orthoslice of onychites marked *x-y* in picture A. (G) Bone fragment. (A–F) PMO 250.281. (G) PMO 250.904.

Inclusions in morphosype D are described by one CT-scan and two thin sections. Morphotype D coprolites are packed with angular inclusions observed in both CT and thin sections. The inclusions vary significantly in shapes and sizes within one specimen, and are interpreted to stem from a variety of prey. The CT-scan of specimen PMO 250.528 stands out from the rest by having inclusions in only half of the specimen, while the remaining part is empty (Fig. 6B). It includes several long, thin inclusions interpreted as possible gill rakers as they have a needle-like shape with a sharp point and slight bend (Fig. 6C). The possible gill rakers measure 3.6 mm and 5.4 mm in length which correlates in both size and shape to Fig. 7, E-I from De Lange et al. (2023).

**Figure 8:**
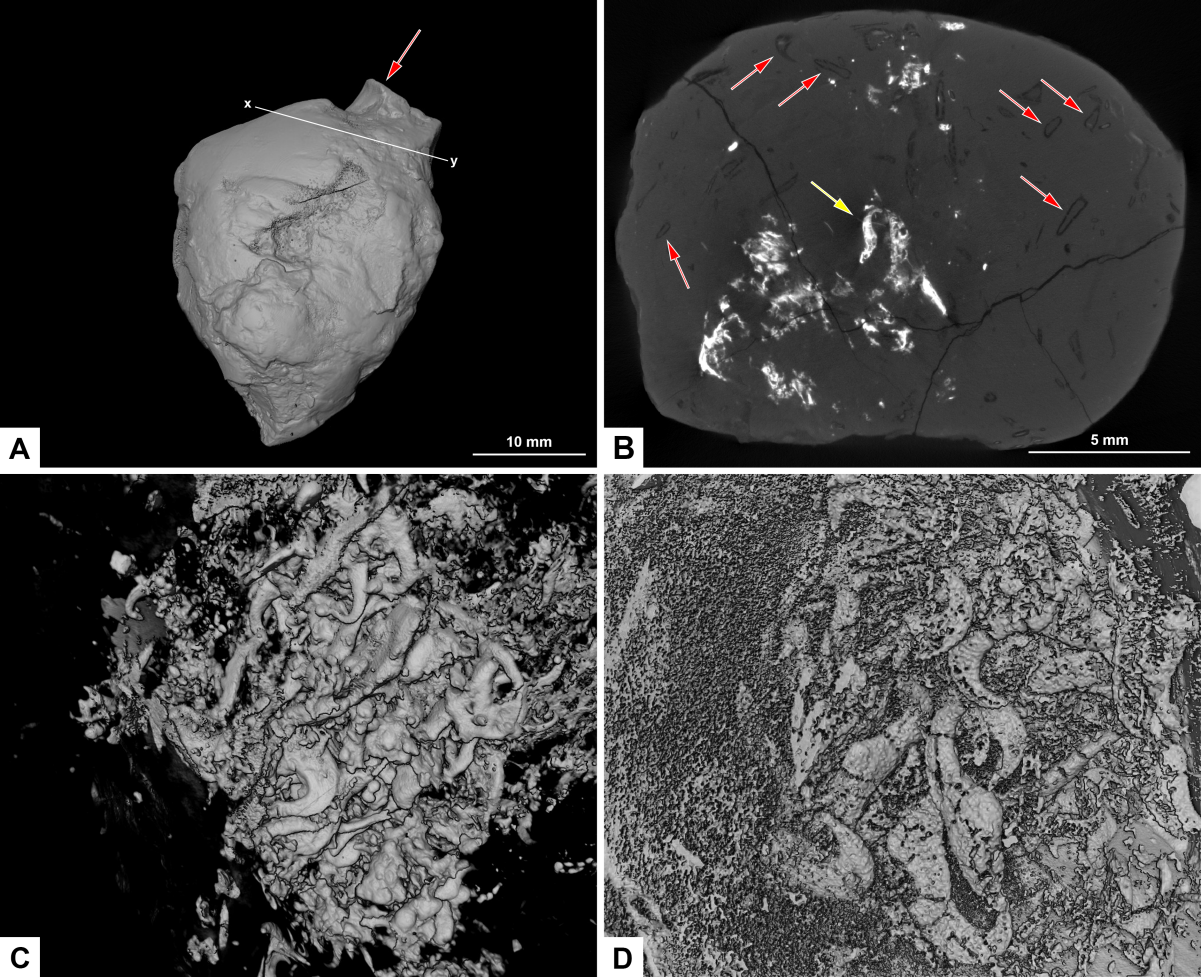
CT-scans of morphotype F, PMO 250.282. (A) External view of morphotype F with x-y being cross section seen in picture B. Red arrow pointing to bone stuck to the surface. (B) Orthoslice from x-y in picture A. Red arrows pointing to onychites with low density, yellow arrow pointing to onychites with high density. (C) Collection of onychites in high density. (D) Inverted CT-reconstruction showing a collection of onychites in low density.

Thin sections show a fine-grained matrix with bones (Fig. 6D), fish scales and secondary mineralized infill after dissolved inclusions in a variety of shapes that are unique for this morphotype (Fig. 6E). SEM/EDS results show that the infill consists of calcite. At least three of the mineralized infills are interpreted to be pseudomorphs after onychites when compared with CT orthoslices from other morphotypes (comparable to PMO 250.282 Fig. 8B). The onychites have a long oval shape with lower density in the middle when seen in orthoslice.

### Morphotype E: wide cylindrical

#### Morphology

Morphotype E (Fig. 7) has a wide, cylindrical shape. The ends are isopolar with flat or concave terminations. The surface is a combination of being smooth and having a wrinkle-like texture.

Many morphotype E coprolites were found as two or more separate pieces that were glued back together.

#### Inclusions

Inclusions of morphotype E are described by one inclusion visible in the hand-held specimen, two CT-scans and two thin sections. This wide cylindrical morphotype has a homogenous matrix in thin section with large angular fragments of bones and cephalopod remains in CT, and small bone fragments in thin sections.

PMO 250.281 is cracked into four pieces and contains an orthoceratoid cephalopod shell fragment, visible in the hand-held specimen (Figs. 7A–7C). The fragment is possibly *Trematoceras* (Hansen et al., 2024). The shell measures 15.8 mm in length. CT-reconstruction of PMO 250.281 shows the cephalopod shell in detail, with visible chambers (Figs. 7D, 7E). The specimen is otherwise homogenous with only a few small bone fragments, as well as a high abundance of onychites in the inverted CT-reconstruction and in orthoslices. In contrast, specimen PMO 250.904 has no cephalopod remains, but does instead contain large angular bone fragments in CT (Fig. 7G). The largest fragment measures 3.73 mm in length.

In thin sections, morphotype E has a fine-grained matrix with small, degraded bone fragments and a few angular fragments.

### Morphotype F: uncategorized (PMO 250.282)

#### Morphology

The overall shape of PMO 250.282 is irregular (Fig. 8). It is closest to being sub-rounded (morphotype C) measuring 26–35 mm in diameter but has an irregular surface with indications of being broken and reworked. Inclusions also differ from morphotype C.

#### Inclusions

A small bone is visible on the surface of the coprolite (Fig. 8A). One CT-scan was made and confirmed that the bone visible at the surface is not inside the coprolite, but is stuck to the exterior surface, and therefore not noted as an inclusion. Orthoslices show that this coprolite is packed with onychites in large collections. The onychites measure approximately 1.5 mm in length and are preserved both with low X-ray density, and high density as interpreted mineralized infill (Fig. 8B), where low density is most common. The CT-reconstructed 3D model as well as orthoslices show a very high abundance of onychites both in high and low density (Figs. 8C, 8D). PMO 250.282 is the coprolite with the highest number of onychites in this study (>80). Similar structures to the onychites in orthoslices can also be seen in the thin section of PMO 250.005 (Figs. 6E, 6F).

### Chemical composition

**Figure 9:**
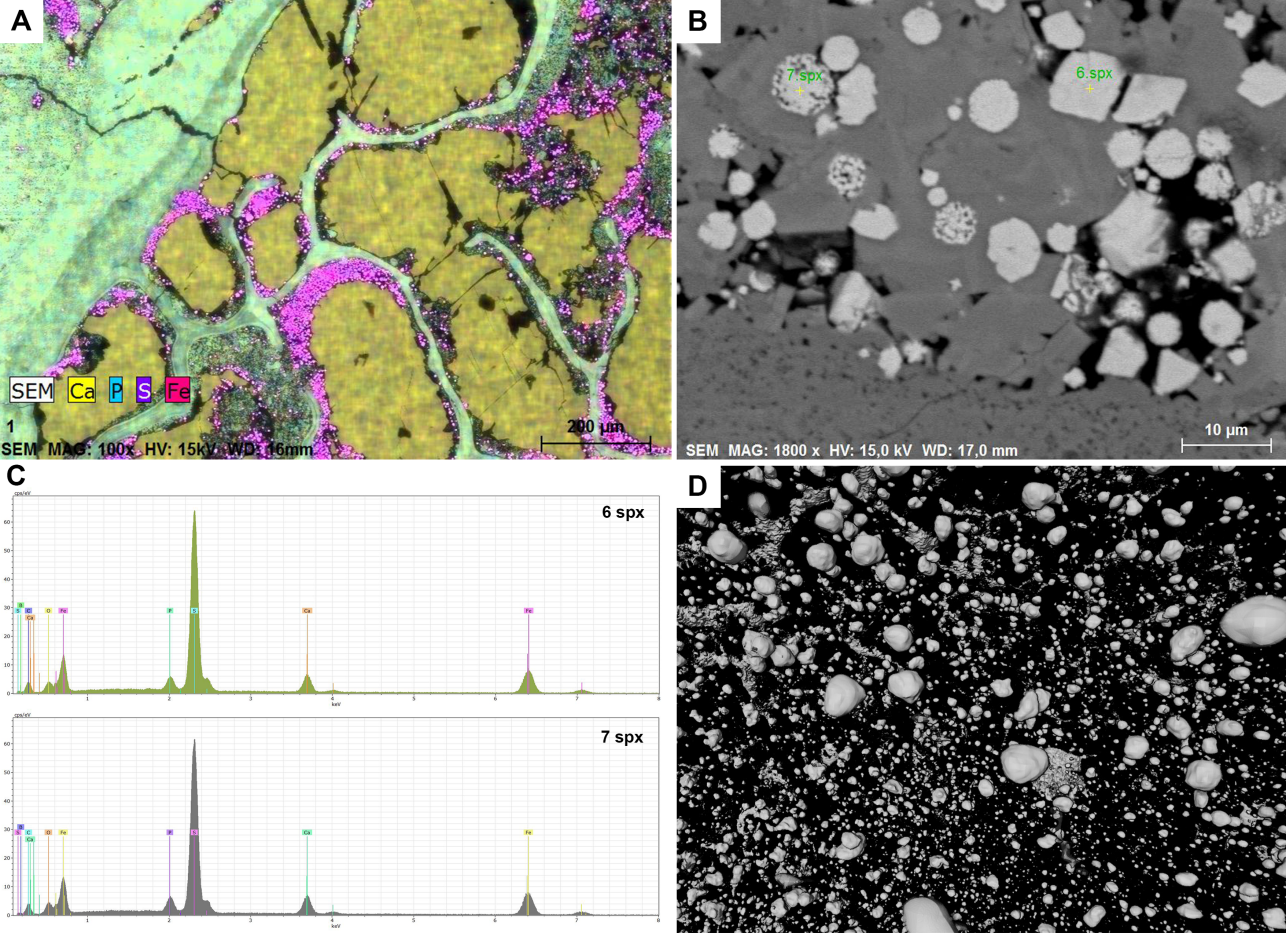
Chemical results. (A) SEM, Elemental map of PMO 250.004. The matrix and the bone consist of calcium phosphate. Pores inside bone cross section are filled with calcium carbonate. Pyrite grows in the outline of inclusion infill. (B) SEM picture including euhedral and framboid pyrite, PMO 250.004. (C) EDS results of measured points seen in picture B, PMO 250.004. (D) Pyrite seen in CT-reconstruction.

Three coprolites from morphotypes amphipolar (A1), sub-rounded (C), and reniform (D) were studied using SEM/EDS (Table S3). Point analyses of all three specimens show a matrix with elevated levels of phosphorus and calcium. A calcium phosphate composition is typical for carnivore coprolites as the phosphate gets derived from the digested bone material, whereas coprolites from herbivores are typically more calcareous (Hunt, Lucas & Spielmann, 2013; Cueille et al., 2020). An elemental map (Fig. 9A) and point analyses (Table S3) was made for PMO 250.004 in an area with matrix as well as a cross section of a possible tetrapod bone (Fig. 9A). The outer parts of the bone have the same calcium phosphate composition as the matrix while pores in the bone have been filled with calcium carbonate (calcite). Calcium carbonate infill is also seen in other morphotypes and seems to be a common feature in the Grippia bonebed coprolites. Another typical feature is the growth of pyrite at the margins of the inclusions.

Pyrite growth is seen in almost every coprolite to varying degrees. SEM imaging shows that there are both euhedral and framboid pyrites (Figs. 9B, 9C) in various sizes. The differences in types of pyrite indicate a dysoxic depositional environment (Shen et al., 2007; Bond & Wignall, 2010). Pyrite grains are also seen in CT scans where they appear more spherical (Fig. 9D), but this may be caused by the resolution of the scans.

### Abundances of inclusions vary between morphotypes

The difference between abundances of inclusions across morphotypes was tested statistically. Inclusions abundance (Table 2) was treated as an ordinal variable where 0 = very low/empty; 1 = medium; 3 = high, scored relatively to each other by looking at the overview internal 3D-models of the coprolites. Medium abundance ranges from approximately 40%–60% of the internal view showing inclusions, while low is <40% and high >60%. The morphotypes were found to have significantly different inclusion abundances (Kruskal-Wallis test, *H* = 17.1, *p* = 0.017). Sample sizes are not sufficient for post-hoc testing to locate these differences formally, but Table 2 indicates that A3, B1, C and E have few inclusions while A1, A2, B2 and D have many inclusions. Further detailed reaserch on this topic are needed.

**Table 2:** Description summary of coprolite morphotypes in the Grippia boned including potential producers.

Morphotype	Very low/empty	Medium	High
A1		1	1
A2		1	2
A3	3		
B1	3		
B2		1	3
C	2	1	
D			1
E	2		

## Discussion

### Are coprolites a reliable source of information?

#### Morphotypes

Defining morphotypes is important for identifying possible producers but is hampered by the reworked and fragmented nature of the material. There is likely a sampling bias toward middle-sized coprolites as the smaller specimens are easier to overlook, whereas the larger specimens are too fragmented to be identified. Triassic marine and aquatic coprolites are commonly between 2–75 mm in length with the marine ones being the smallest (Jain, 1983; Brachaniec et al., 2015; Cueille et al., 2020; Mingtao et al., 2022), whereas the Grippia bonebed coprolites are 7–40 mm in general. Two specimens are larger with a maximum length of 95 mm. The two larger specimens imply that larger coprolites do exist but are rarely preserved in their complete form. The bias towards medium sizes is, therefore, thought to be a result of preservation and fragmentation.

Taphonomic processes may alter the morphology of the coprolites. It is possible that sub-morphotype A1 is a reworked and polished variant of A2 as they exhibit a smooth and polished surface. Another explanation may be that when spiral shaped coprolites get deposited in water, the spiral will loosen and uncoil over time until they get buried (Jain, 1983).

Loss of spiral structure possibly happened with sub-morphotype A2 as the resemblance to uncoiling coprolites in Fig. 7 from Jain (1983) is noteworthy. Some A1 specimens may also resemble A3 coprolites that have been reworked and polished, but differences in inclusions suggest that they are distinct.

As within morphotype A, it is possible that sub-morphotype B2 is a reworked and polished version of sub-morphotype B1. However, the difference in inclusions strongly suggests that they are distinct (Table 2) and therefore suggest that they are produced by different animals. The differences may still be caused by the same animal group, with species or yearly variations in diets and slightly different shapes.

Spiral coprolites are commonly grouped together in the same morphotype but are separated in this study. All shapes from the straight sub-morphotype A1 to the spiraling A2 coprolites have been found and are often hard to separate from each other indicating gradual reworking of the same morphotype. Sub-morphotype B2 differs from A2 by being more rounded like B1 and exhibiting heteropolar spiraling, as opposed to amphipolar spiraling observed in A2. While the current morphotype classification system may be changed to combine both spiral coprolites in the future, this system is considered the most practical approach for this study.

Both morphotype B and E exhibit flat to concave terminations indicating that they have been pinched off during extrusion or broke into smaller pieces while fresh (Milàn, 2012; Dentzien-Dias et al., 2020). It is therefore impossible to know the true size and spiral type of these specimens. Some of the B2 and E coprolites might even be segments of the same morphotype, where B2 would be the end pieces and E the middle segments (Milàn, 2012, fig. 3; Milàn et al., 2021). PMO 250.281 from morphotype E resembles a longer segment and suggests that the morphotype has a slight sinuous or bumpy surface structure.

#### Inclusions

Inclusions can provide a better understanding of diet, but the degree of digestion and preservation still needs to be considered. Fish scales containing ganoine have a better preservation potential than other bone material (Richter & Smith, 1995). Bone material varies from lightly to severely digested due to bones being more fragile and differences in the efficiency of the digestive system of the host animal (Bajdek et al., 2017). Invertebrates are the least preserved inclusion type and are only preserved in the Grippia bonebed coprolites if they exhibit mineralized hard parts as for example shell or silika spicules.

Sponge spicules have been found inside one entire coprolite specimen (PMO 250.265), suggesting that they were ingested rather than attached to the surface by contact with the sediment. Alternatively, the spicules could have been swallowed by accident by a scavenger or durophagous animal feeding on something off a spicule filled sediment. However, two pieces of sediment from the bonebed were CT-scanned to look for spicules, and none were found. It is therefore not likely that the Grippia bonebed sediment included spicules, although spicules have been found lower in the Vikinghøgda Formation (Foster et al., 2023). PMO 250.265 may have been produced by an animal that had ingested sponges at another locality, and then excreted feces which eventually were deposited in Grippia bonebed. Alternatively, the lack of spicules in the sediment may be the result of reworking and diagenesis.

Due to their low X-ray density, onychites are for the most part only visible in orthoslices and in inverted CT-scan 3D reconstructions. In a normal 3D-reconstruction it is possible to see zero to three onychites within each specimen, whereas the inverted model and orthoslice reveal up to 80 onychites in the same coprolites. Specimens PMO 250.005 and PMO 250.282 contain a few mineralized onychites and show that they can be preserved in two different ways; in high or low-density materials (Figs. 6E, 8B–8D).

The coprolites are very brittle and cracked. The cracks may have exposed parts of the coprolite to different types of surrounding fluids and bacteria that could have affected the type of preservation. Exactly how different types of preservation happened needs more research looking into biological, chemical, and physical factors.

Overall, the Grippia bonebed coprolites show a close relationship between the morphotypes, and amount and type of inclusions within each morphotype (Table 2) indicating that they are produced by different animal groups. There are many factors to take into consideration when analyzing coprolites and it is important to be aware of the uncertainties in deformation of morphotypes and potentially missing inclusions. The inclusions that we do see are, however, a good source of information and show which animals were preyed on. Conversely. the lack of inclusions can still be used as an indicator for a slow metabolism, a diet of soft-bodied animals or that the animal regurgitated some of its prey (Pollard, 1968; Gordon et al., 2020).

### Grippia bonebed coprolites highlight ecological interactions

The Grippia bonebed contains fossils of, ichthyopterygians, ichthyosauriforms, temnospondyls, chondrichthyans, dipnoans, sarcopterygians and actinopterygians (Bratvold, Delsett & Hurum, 2018). The coprolites were most likely produced by these animals. Morphotypes also vary slightly within each animal group (Fisher, 1981), and one species can produce different looking feces depending on their prey and feces plasticity (Williams, 1972).

It is therefore not sufficient to only use the morphotypes, but combining information about the animal’s diet, behavior, and association with skeletal material in combination with characteristic morphotypes can be helpful (Hunt & Lucas, 2010; Jurigan, Ricardi-Branco & Dentzien-Diaz, 2023).

The phosphatic matrix and animal remains inclusions indicate that the coprolites are likely produced by carnivores (Hunt, Lucas & Spielmann, 2013; Cueille et al., 2020). The size of the coprolites indicates the source to be vertebrates, as invertebrate coprolites would be less than five mm (Dridi, 2022).

Based on the assumption that sub-morphotype A1 originally was amphipolar just like A2, it is likely that they were produced by non-teleost fish that are known to have a simple spiral valve. In contrast to chondrichthyans who are known to produce heteropolar spirals (McAllister, 1985; Williams, 1972). The Grippia bonebed contains remains of large coelacanths, predatory actinopterygian fishes like *Saurichthys and Birgeria*, and rare remains of dipnoans. All these fishes are possible producers of amphipolar coprolites (Williams, 1972; Jain, 1983; Hunt & Lucas, 2012; Argyriou et al., 2016). Lungfish such as *Ceratodus* are less likely because they are rare in the bonebed, and typically produce coprolites with a fine-grained matrix and a lack of inclusions due to them being omnivores, mostly feeding on plants and soft bodied animals (Argyriou et al., 2016; Cueille et al., 2020). *Saurichthys* cololites also usually lack recognizable bone remains (Argyriou et al., 2016). This leaves coelacanths to be the most likely producer of amphipolar coprolites that are packed with inclusions, whereas other predatory fish like *Saurichthys* or *Birgeria* might have produced the amphipolar coprolites with fewer inclusions and well digested bones. The possible ichthyopterygian bone from PMO 250.004 indicates that these large predatory fishes were feeding on everything from other fish to juvenile ichthyopterygians.

Coprolites with striations and grooves, like sub-morphotype A3, are typical for reptiles (Krause & Piña, 2012; Young, 1964), and especially for modern crocodilians and extinct archosauromorphs (Young, 1964; Northwood, 2005; Milàn & Hedegaard, 2010; Milàn, 2012). Sub-morphotype A3 is a perfect example of archosauromorph coprolites with its elongated shape, tapered ends, constriction marks, and homogenous matrix. The few small inclusions including teeth and conodont elements are comparable to fragments seen in modern crocodilians. Other than enamel less teeth remains, crocodilian feces are generally devoid of bone remains due to their highly concentrated stomach acid (Fisher, 1981; Milàn, 2012). PMO 250.267 and PMO 250.273 may have been produced by a larger reptile than the rest of sub-morphotype A3. The diameter of extant crocodilian feces has been proven to correlate with the total length of the animal (Milàn, 2012). Motani (2000) hints to an animal, possibly large reptile from the Grippia niveau. As the A3 coprolites are similar to known archosauromorph coprolites, they may be their producers. The largest A3 specimens measure 23 mm and 45 mm in diameter, which correlates to an animal with a body length of around 1–2.5 m. The measurements correlate with the possible archosauromorph skeletal material described by Motani (2000), but the possible shrinkage during dehydration and diagenesis makes this calculation conservative.

Modern crocodilians often have a slight bend to their feces similar to morphotype D from the Grippia bonebed (Young, 1964; Milàn, 2012; Krause & Piña, 2012). However, morphotype D coprolites have a considerable number of inclusions (bones, fish scales and onychites) making it unlikely that they were produced by archosauromorphs. Instead, the high number of varied inclusions suggests an opportunistic or scavenging animal. Cylindrical feces are common for all tetrapods, therefore, the possible producers might be temnospondyls (Northwood, 2005).

Rounded spiral coprolites (B1) typically with a heteropolar spiral, as seen in the Grippia bonebed, are generally accepted to be produced by sharks (Williams, 1972; McAllister, 1985; Dentzien-Dias et al., 2012). As modern shark feces are mostly liquid (Williams, 1972) there might be a preservation bias for hybodont shark coprolites that were possibly originally more solid. Hybodonts are the most common shark teeth in the Grippia bonebed (Bratvold, Delsett & Hurum, 2018), and with the coprolites containing fish remains, hybodont sharks are likely producers. Sub-morphotype B1 has slight differences in the overall roundness, length and termination types. Morphological differences suggest that these coprolites may be produced by different, but closely related shark species (Mingtao et al., 2022). Rounded, spiral coprolites are rare in the Grippia bonebed, supporting the idea that they fossilized less easily. Alternatively, the spiral structure might have been reworked and polished, making it difficult to identify this morphotype. For discussion on chondrichthyans in the Grippia bonebed see Bratvold, Delsett & Hurum (2018) and Saugen et al. (2025).

Sub-morphotype B2 differs significantly from B1 by having a high abundance of inclusions, as well as including onychites and sponge spicules. The variation in inclusions indicates differences in the efficiency of the digestive system, or a very different diet, and therefore, a different producer than B1. Teardrop coprolites have earlier been assigned to teleost fish (Jurigan, Ricardi-Branco & Dentzien-Diaz, 2023). The producer of PMO 250.265 ingested sponges. It is common for tropical fish today to feed on sponges, but the fish needs to have specialized dentition to do so (Verdín Padilla, Carballo & Camacho, 2010). From the Grippia bonebed, *Bobasatrania* is the most likely fish to eat sponges due to tooth morphology. However, the specimen (PMO 250.265) also contains bone material and onychites in addition to the sponge spicules. It is therefore more likely that the coprolite was produced by scavenging or demersal feeding accidentally ingesting spicules either from its prey, or from sediment at another locality. The B2 teardrop morphotype may have been produced by different types of osteichthyans, except for coelacanths, dipnoans and *Saurichthys* that are known to produce amphipolar coprolites (Argyriou et al., 2016).

When it comes to sub-rounded coprolites (morphotype C) it is more difficult to suggest any producers. The morphotype has earlier been associated with temnospondyls (Niedźwiedzki et al., 2016a), but also with durophagous fish (Cueille et al., 2020; Dridi, 2022). The Grippia bonebed sub-rounded coprolites were produced by an animal with a relatively efficient digestive system, that swallowed large pieces of food. No specific producer can be assigned to this morphotype in the Grippia bonebed as most animals can produce this shape as part of a larger fecal mass.

Morphotype E has the widest diameter of all the morphotypes, which indicates a large animal, likely reptilian because of their cylindrical shape (Young, 1964; Thulborn, 1991; Krause & Piña, 2012). Inclusions of relatively large bone fragments suggest that this animal swallowed large pieces of food. Among the larger reptiles in the bonebed, ichthyopterygians and archosauromorphs are possible candidates. Archosauromorphs have been associated with segmented coprolites (Milàn, 2012; Bajdek et al., 2017). If morphotype E was produced by archosauromorphs as likely morphotype A3, they should be homogenous. However, morphotype E has larger and more inclusions indicating that this animal had a different digestive system efficiency. Studies of the bone of ichthyosaurs indicate that they had a high metabolic rate (Sander, 2021). Morphotype E usually has few large bone inclusions and an abundance of onychites. Earlier studies on ichthyosaurs (Pollard, 1968; Lomax, 2010) found that hooks accumulated in the stomach, showing that cephalopods were important prey. Ichthyopterygians would be more likely to catch a lot of cephalopods as they are considered to have been active predators (Pollard, 1968) compared to archosauromorphs, A study looking at the stomach contents of an Early Triassic ichthyosaur from Spitsbergen also found cephalopod hooks (Buchy, Taugourdeau & Janvier, 2004). It has been discussed if ichthyosaurs may have regurgitated the material from time to time, and that it did not go through the digestive tract at all, as sperm whales do today (Pollard, 1968). However, it is possible that some of the hooks may have made their way through. Ichthyosaurs may also have been able to only bite off the soft parts of its prey (Klug et al., 2021), however this does not explain the shell fragment found in PMO 250.281. Jurassic ichthyosaurs were able to remove the hard parts of cephalopod prey (Klug et al., 2021). The Grippia bonebed coprolites suggest the possibility that early ichthyopterygians were also specialized to eat cephalopods but may be less successful removing the hard parts. Ichthyosaur coprolites are not well known. The lack of known ichthyosaur coprolites may be explained by comparing them with living whales and sharks which have fecal matter resembling liquid clouds, lacking solid material (Williams, 1972). Hunt, Lucas & Spielmann (2012) suggested that coiled cylindrical coprolites may belong to ichthyosaurs, however no tetrapods that we know of have a spiral intestine (Northwood, 2005). The ichnotaxon *Ichthyosaurolites duffini* (Hunt & Lucas, 2012) describes a rectangular segmented coprolite with angular inclusions that closely resembles morphotype E from the Grippia bonebed. Combining the wide cylindrical shape with the state and type of inclusions makes ichthyopterygians like *Grippia*, *Omphalosaurus* or *Cymbospondylus* the most likely producers.

#### Invertebrates

The discovery of invertebrate remains in the coprolites is the first evidence of invertebrates in the Grippia bonebed. Further, sponge spicules are the first evidence of benthic organisms, and the first evidence of an animal feeding on benthic organisms in this area. Hansen et al. (2024) found almost no benthic invertebrates in the lower Vendomdalen Member, becoming more abundant in the upper part.

Cephalopod arm hooks (onychites) are found in morphotypes A2, B2, D, E and F. The number of onychites suggests that cephalopods were a common prey for the active predators in the Early Triassic Boreal Sea. Scattered belemnoid onychites as well as orthoceratoids and ammonoids have been reported from several levels in the Vendomdalen Member (Hansen et al., 2024). Most coprolites contain a variety of prey, while the producer of morphotype E may have had cephalopods as their main prey. In cases where only a few onychites were found, it may be that the producer swallowed them accidentally when ingesting a prey that had eaten a cephalopod.

Evidence of coprophagy is found in two coprolite specimens as possible burrows and borings (PMO 250.530 and PMO 250.271). Burrows inside PMO 250.530 are made parallel to the surface and have turns indicating that this pattern was made by a living organism and not just by escape of gas bubbles formed during decomposition (Wahl, Martin & Hasiotis, 1998). PMO 250.530 has burrows with no entry or exit points, likely made when the coprolite was still soft and buried rapidly. These could also be caused by organisms from digestive tract itself, such as nematodes. Nematodes have been found in marine coprolites from the Lower Triassic of Poland and may occur inside the digestive tract of marine animals (Brachaniec et al., 2015). PMO 250.271 has traces that differ from the internal burrows in PMO 250.530. The specimen has borings made from the outside going inward perpendicular to the surface, indicating the presence of unknown organisms in the Grippa bonebed. Borings are well known in the coprolite record, and are likely made after phosphatization as the entry points are still open (Tapanila et al., 2008; Milàn, Rasmussen & Bonde, 2012; Dentzien-Dias et al., 2018; Dentzien-Dias et al., 2020).

## Conclusions

The Grippia bonebed coprolites provides new insights into the paleoecosystem and ecological interactions of the Early Triassic. The eight sub-morphotypes described could be tentatively assigned to likely vertebrate producers comparing with abundant skeletal remains in the bonebed. The shapes of the coprolites are altered by poor preservation, fragmentation and reworking which makes a comparison with inclusions important to identify possible producers. Inclusions indicate that fish were the most common prey, but larger predators were also feeding on juvenile ichthyopterygians. Notably, the coprolites include the first known remains of invertebrates in the Grippa bonebed such as cephalopods and sponges. The coprolites also record traces of coprophagy. Lastly, inverting the CT-reconstructions proved to be highly effective for identifying cephalopod hooks and will be a valuable technique for future studies.

##  Supplemental Information

10.7717/peerj.20746/supp-1 Supplemental Information 1 Settings used for CT-scanning each coprolite specimen

10.7717/peerj.20746/supp-2 Supplemental Information 2 List of PMO (museum numbers) for all coprolite specimens in this study and what they were used for

10.7717/peerj.20746/supp-3 Supplemental Information 3 SEM/EDS results of coprolites in atomic weight percentage

10.7717/peerj.20746/supp-4 Supplemental Information 4 Morphotype A3 largest specimen with typical grooves PMO 250.267 measuring 95 mm in length and 45 mm in width.

10.7717/peerj.20746/supp-5 Supplemental Information 5 Additional CT-scans (A-C) and thin sections (D) of morphotype A (A) Possible shark tooth measuring 0,45 x 0.5 mm, PMO 250.273. (B) Articulated fish tail, PMO 250.279. (C) Possible neural spine measuring 4,7 mm in length, PMO 250.267. (D) Fish scale with enamel and growth lines, PMO 250.004.

## Additional Information and Declarations

### Field Study Permissions

The following information was supplied relating to field study approvals (*i.e.*, approving body and any reference numbers): All necessary excavation permits were obtained from the Governor of Svalbard permit no 2013/01222-2.

### Data Availability

The following information was supplied regarding data availability: The CT data are available at Morphosorce: PMO 250.270 3D model (ID 000780372). https://doi.org/10.17602/M2/M780372
PMO 250.270 (ID 000723138). https://doi.org/10.17602/M2/M723138
PMO 250.275 3D model (ID 000780366). https://doi.org/10.17602/M2/M780366
PMO 250.275 (ID 00075070). https://doi.org/10.17602/M2/M725070
PMO 250.273 3D model (ID 000780356). https://doi.org/10.17602/M2/M780356
PMO 250.273 (ID 000733962). https://doi.org/10.17602/M2/M733962
PMO 250.533 3D model (ID 000780229). https://doi.org/10.17602/M2/M780229
PMO 250.533 (ID 000729921). https://doi.org/10.17602/M2/M729921
PMO 250.265 3D model (ID 000780217). https://doi.org/10.17602/M2/M780217
PMO 250.265 (ID 000733979). https://doi.org/10.17602/M2/M733979
PMO 250.271 3D model (ID 000780194). https://doi.org/10.17602/M2/M780194
PMO 250.271 (ID 000731949). https://doi.org/10.17602/M2/M731949
PMO 250.528 3D model (ID 000780189). https://doi.org/10.17602/M2/M780189
PMO 250.528 (ID 000729931). https://doi.org/10.17602/M2/M729931
PMO 250.282 3D model (000780183). https://doi.org/10.17602/M2/M780183
PMO 250.282 (ID 000738344). https://doi.org/10.17602/M2/M738344
PMO 250.285 (ID 0007393181). https://doi.org/10.17602/M2/M739181
PMO 250.536 (ID 000739119). https://doi.org/10.17602/M2/M739119
PMO 250.535 (ID000737411). https://doi.org/10.17602/M2/M737411
PMO 250.534 (ID 000736987). https://doi.org/10.17602/M2/M736987
PMO 250.281 (ID 000736804). https://doi.org/10.17602/M2/M736804
PMO 250.267 (ID 000736181). https://doi.org/10.17602/M2/M736181
PMO 250.904 (ID 000736058). https://doi.org/10.17602/M2/M736058
PMO 250.277 (ID 000735719). https://doi.org/10.17602/M2/M735719
PMO 250.841 (ID 000734401). https://doi.org/10.17602/M2/M734401
PMO 250.532 (ID 000729936). https://doi.org/10.17602/M2/M729936
PMO 250.530 (ID 000729926). https://doi.org/10.17602/M2/M729926
PMO 250.284 (ID 00075129). https://doi.org/10.17602/M2/M725129
PMO 250.531 (ID 000724403). https://doi.org/10.17602/M2/M724403
PMO 250.279 (ID 000724390). https://doi.org/10.17602/M2/M724390
PMO 250.529 (ID 000723715). https://doi.org/10.17602/M2/M723715.
