# Supplementary material for: Digesting an ancient ecosystem: coprolites from the Grippia bonebed, Lower Triassic, Svalbard
Source: PeerJ. 2026 Feb 17;14:e20746. doi: 10.7717/peerj.20746 (PMC12922587; doi:10.7717/peerj.20746)
Supplement: Supplemental Information 1 [file peerj-14-20746-s001.docx]

| PMO | Projections | Frames | Exposure (ms) | Voxel size (mm3) | Filter material | x-ray kV | x-ray uA |
| --- | --- | --- | --- | --- | --- | --- | --- |
| 250.265 | 3343 | 1 | 1000 | 0,0090 | Aluminium | 93 | 121 |
| 250.267 | 4081 | 2 | 1000 | 0,0364 | Tin | 112 | 135 |
| 250.270 | 2523 | 1 | 1000 | 0,0110 | Aluminium | 90 | 114 |
| 250.271 | 4523 | 4 | 1000 | 0.0058 | Aluminium | 94 | 120 |
| 250.273 | 2450 | 1 | 1000 | 0,0248 | None | 95 | 124 |
| 250.275 | 2808 | 1 | 1000 | 0,0124 | Aluminium | 88 | 129 |
| 250.277 | 4102 | 1 | 1000 | 0,0182 | Aluminium | 102 | 117 |
| 250.279 | 4166 | 1 | 1000 | 0,0121 | Aluminium | 101 | 117 |
| 250.281 | 3439 | 2 | 1000 | 0,0184 | None | 110 | 140 |
| 250.282 | 4523 | 2 | 1000 | 0,0139 | None | 90 | 145 |
| 250.284 | 3424 | 1 | 1000 | 0,0122 | Aluminium | 99 | 120 |
| 250.285 | 3764 | 1 | 1000 | 0,0080 | Aluminium | 91 | 118 |
| 250.528 | 1868 | 2 | 1000 | 0,0216 | Tin | 134 | 136 |
| 250.529 | 1836 | 2 | 1000 | 0,0216 | Tin | 134 | 136 |
| 250.530 | 1514 | 1 | 1000 | 0,0225 | Tin | 113 | 137 |
| 250.531 | 1450 | 1 | 1000 | 0,0394 | Tin | 110 | 135 |
| 250.532 | 1489 | 1 | 1000 | 0,0286 | Tin | 110 | 135 |
| 250.533 | 2097 | 1 | 1000 | 0,0255 | None | 110 | 135 |
| 250.534 | 2883 | 4 | 500 | 0,0071 | None | 85 | 370 |
| 250.535 | 3423 | 4 | 500 | 0,0080 | None | 85 | 370 |
| 250.536 | 3721 | 2 | 1000 | 0,0063 | Aluminium | 75 | 250 |
| 250.841 | 4519 | 2 | 1000 | 0,0058 | Aluminium | 90 | 205 |
| 250.904 | 3862 | 2 | 1000 | 0,0181 | Tin | 110 | 135 |
