# Supplementary material for: Digesting an ancient ecosystem: coprolites from the Grippia bonebed, Lower Triassic, Svalbard
Source: PeerJ. 2026 Feb 17;14:e20746. doi: 10.7717/peerj.20746 (PMC12922587; doi:10.7717/peerj.20746)
Supplement: Supplemental Information 2 [file peerj-14-20746-s002.docx]

| Morphotype | PMO | Length*width (mm) | CT scan | Thin section | Photographed | Figure |
| --- | --- | --- | --- | --- | --- | --- |
| A1 | 250.004 | 17 x 6 |  | x | x | 3B, 9A-C, S2D |
|  | 250.006 | 26 x 6 |  | x | x |  |
|  | 250.007 | 25 x 6 |  | x | x |  |
|  | 250.008 | 23 x 5 |  | x | x |  |
|  | 250.009 | 15 x 4 |  | x | x | 3C |
|  | 250.270 | 23 x 6 | x |  |  | 3A |
|  | 250.529 | 25 x 7 | x |  |  |  |
|  | 250.842 | 25 x 7 |  |  |  |  |
|  | 250.843 | 18 x 5 |  |  |  |  |
|  | 250.844 | 16 x 3 |  |  |  |  |
|  | 250.845 | 35 x 6 |  |  | x |  |
|  | 250.846 | 16 x 4 |  |  | x | 2B |
|  | 250.847 | 22 x 5 |  |  | x | 2A |
|  | 250.848 | 11 x 4 |  |  |  |  |
|  | 250.849 | 15 x 5 |  |  |  |  |
| A2 | 249.999 | 24 x 6 |  | x | x | 3E |
|  | 250.000 | 23 x 7 |  | x | x | 3F |
|  | 250.275 | 27 x 7 | x |  | x | 2C. 3D |
|  | 250.279 | 34 x 10 | x |  |  | S2B |
|  | 250.284 | 29 x 10 | x |  |  |  |
|  | 250.850 | 18 x 5 |  |  |  |  |
|  | 250.851 | 19 x 7 |  |  |  |  |
|  | 250.852 | 20 x 8 |  |  |  |  |
|  | 250.853 | 6 x 3 |  |  |  |  |
|  | 250.854 | 20 x 6 |  |  | x | 2D |
|  | 250.855 | 28 x 7 |  |  |  |  |
|  | 250.856 | 29 x 7 |  |  |  |  |
|  | 250.857 | 28 x 6 |  |  |  |  |
|  | 250.858 | 30 x 9 |  |  | x | 2E |
| A3 | 250.273 | 64 x 23 | x |  |  | 3G, 3I, S2A |
|  | 250.267 | 95 x 45 | x |  | x | S1, S2C |
|  | 250.530 | 15 x 8 | x |  | x | 2G, 3H |
|  | 250.531 | 32 x 11 | x |  |  |  |
|  | 250.859 | 17 x 5 |  |  |  |  |
|  | 250.860 | 19 x 7 |  |  | x | 2F |
|  | 250.861 | 29 x 5 |  |  |  |  |
| B1 | 250.532 | 29 x 11 | x |  |  |  |
|  | 250.533 | 22 x 17 | x |  | x | 2H, 4A |
|  | 250.841 | 14 x 8 | x |  | x | 2J, 4B |
|  | 250.862 | 9 x 7 |  |  |  |  |
|  | 250.863 | 16 x 11 |  |  |  |  |
|  | 250.864 | 22 x 10 |  |  | x | 2I |
|  | 250.865 | 21 x 13 |  |  |  |  |
|  | 250.866 | 17 x 10 |  |  |  |  |
| B2 | 250.010 | 25 x 11 |  | x | x |  |
|  | 250.265 | 19 x 9 | x |  |  | 4D-F |
|  | 250.534 | 18 x 7 | x |  |  |  |
|  | 250.535 | 19 x 9 | x |  |  |  |
|  | 250.536 | 11 x 7 | x |  | x |  |
|  | 250.868 | 15 x 9 |  |  | x | 2L |
|  | 250.869 | 13 x 7 |  |  | x | 2K |
|  | 250.870 | 14 x 10 |  |  |  |  |
|  | 250.871 | 7 x 4 |  |  |  |  |
|  | 250.872 | 11 x 8 |  |  |  |  |
|  | 250.873 | 16 x 8 |  |  |  |  |
|  | 250.874 | 13 x 8 |  |  |  |  |
|  | 250.875 | 19 x 8 |  |  |  |  |
|  | 250.876 | 15 x 8 |  |  | x |  |
| C | 250.002 | 7 x 8 |  | x | x | 5E |
|  | 250.003 | 16 x 15 |  | x | x |  |
|  | 250.017 | 8 x 7 |  | x | x |  |
|  | 250.018 | 13 x 10 |  | x | x | 5F |
|  | 250.271 | 14 x 11 | x |  | x | 2M, 5A, D |
|  | 250.277 | 40 x 27 | x |  |  | 2B-C |
|  | 250.285 | 14 x 12 | x |  |  |  |
|  | 250.877 | 17 x 13 |  |  |  |  |
|  | 250.878 | 18 x 15 |  |  |  |  |
|  | 250.879 | 6 x 5 |  |  |  |  |
|  | 250.880 | 10 x 10 |  |  |  |  |
|  | 250.881 | 11 x 9 |  |  |  |  |
|  | 250.882 | 7 x 6 |  |  |  |  |
|  | 250.883 | 21 x 19 |  |  |  |  |
|  | 250.884 | 19 x 15 |  |  | x | 2N |
| D | 250.005 | 16 x 6 |  | x | x | 6D-F |
|  | 250.011 | 27 x 19 |  | x | x |  |
|  | 250.528 | 29 x 10 | x |  | x | 2P, 6A-C |
|  | 250.885 | 19 x 7 |  |  |  |  |
|  | 250.886 | 21 x 9 |  |  | x | 2O |
|  | 250.887 | 15 x 7 |  |  |  |  |
|  | 250.888 | 13 x 6 |  |  | x |  |
|  | 250.889 | 15 x 7 |  |  |  |  |
|  | 250.890 | 21 x 10 |  |  |  |  |
|  | 250.891 | 13 x 6 |  |  |  |  |
| E | 250.015 | 32 x 19 |  | x | x |  |
|  | 250.016 | 31 x 26 |  | x | x |  |
|  | 250.281 | 40 x 20 | x |  | x | 2S, 7A-F |
|  | 250.892 | 24 x 20 |  |  |  |  |
|  | 250.893 | 26 x 17 |  |  |  |  |
|  | 250.894 | 15 x 15 |  |  |  |  |
|  | 250.895 | 14 x 19 |  |  |  |  |
|  | 250.896 | 37 x 17 |  |  |  |  |
|  | 250.897 | 12 x 13 |  |  | x | 2R |
|  | 250.898 | 20 x 19 |  |  |  |  |
|  | 250.899 | 29 x 20 |  |  | x | 2Q |
|  | 250.904 | 30 x 20 | x |  |  | 7G |
|  | 250.906 | 32 x 27 |  |  |  |  |
| Unidentified | 250.282 | 35 x 26 | x |  |  | 8A-D |
| Broken | 250.900 | 18 x 23 |  |  |  |  |
|  | 250.901 | 22 x 20 |  |  | x |  |
|  | 250.902 | 19 x 15 |  |  | x |  |
|  | 250.903 | 14 x 15 |  |  |  |  |
|  | 250.905 | 10 x 9 |  |  |  |  |
