# Supplementary material for: Digesting an ancient ecosystem: coprolites from the Grippia bonebed, Lower Triassic, Svalbard
Source: PeerJ. 2026 Feb 17;14:e20746. doi: 10.7717/peerj.20746 (PMC12922587; doi:10.7717/peerj.20746)
Supplement: Supplemental Information 3 [file peerj-14-20746-s003.docx]

| PMO | Placement | C | O | F | P | S | Ca |
| --- | --- | --- | --- | --- | --- | --- | --- |
| 250.002 | Matrix | 9,78 | 54,87 | 5,33 | 8,81 | 0,21 | 20,60 |
| 250.004 | Matrix | 20,91 | 49,42 | 5,43 | 7,12 | 0,47 | 16,66 |
| 250.004 | Bone | 17,36 | 50,86 | 4,86 | 7,74 | 0,36 | 18,43 |
| 250.004 | Infill | 25,51 | 56,53 |  | 0,55 | 0,18 | 17,04 |
| 250.005 | Matrix | 24,02 | 44,89 | 3,97 | 8,18 | 0,26 | 18,40 |
