# Supplementary figures and images for: Digesting an ancient ecosystem: coprolites from the Grippia bonebed, Lower Triassic, Svalbard
Source: PeerJ. 2026 Feb 17;14:e20746. doi: 10.7717/peerj.20746 (PMC12922587; doi:10.7717/peerj.20746)

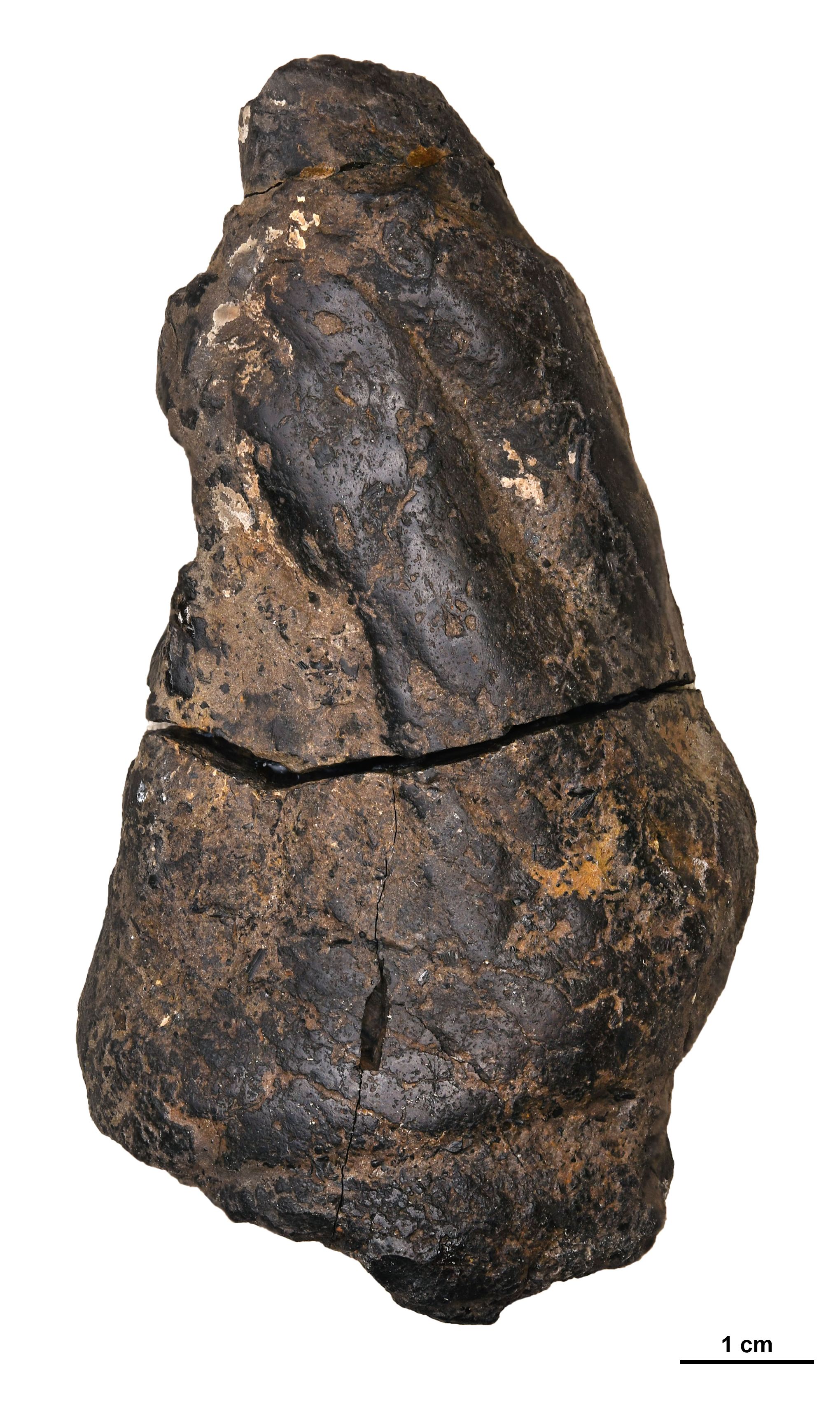

Supplement: Supplemental Information 4 — PMO 250.267 measuring 95 mm in length and 45 mm in width. [file peerj-14-20746-s004.png]

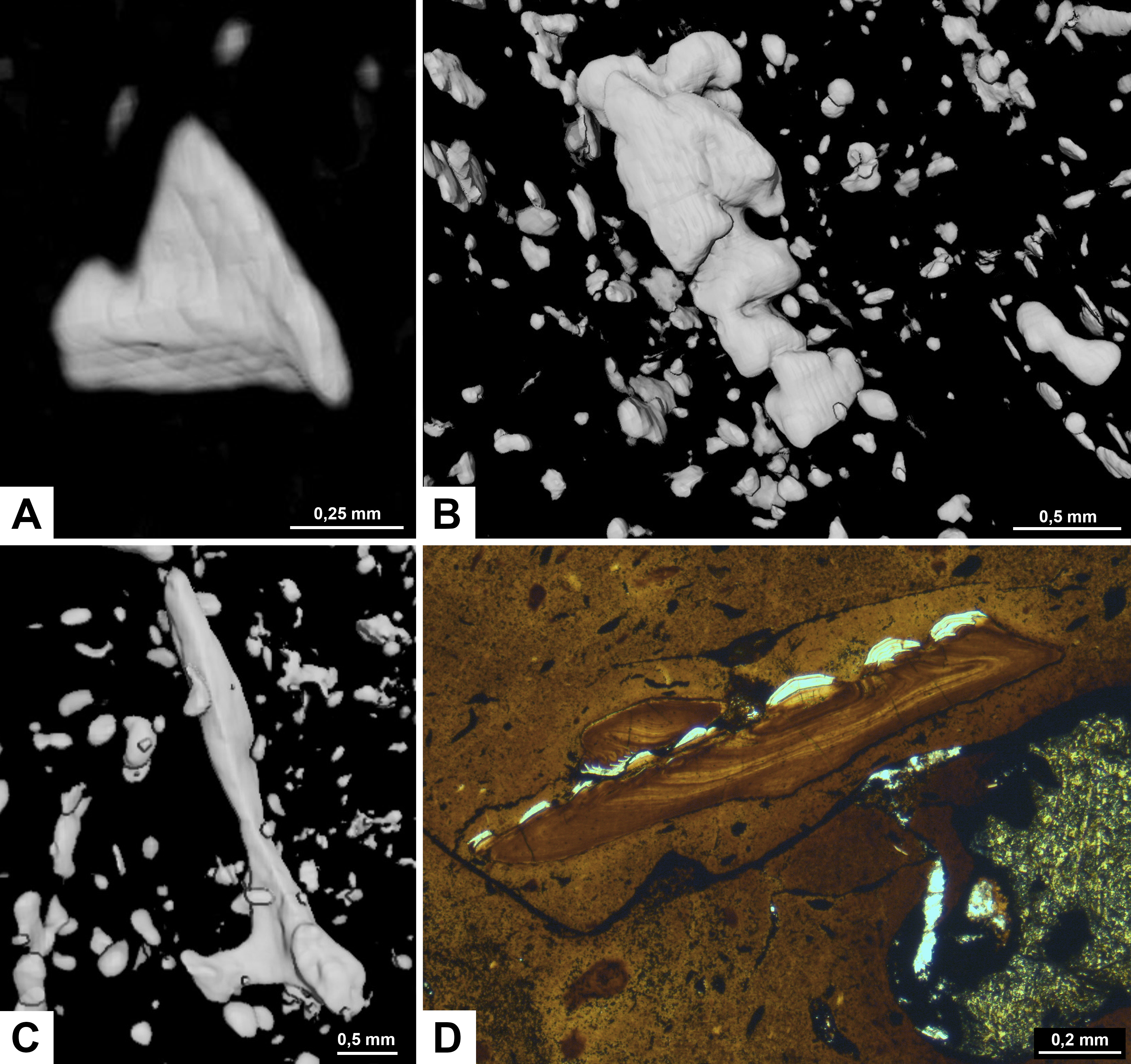

Supplement: Supplemental Information 5 — (A) Possible shark tooth measuring 0,45 x 0.5 mm, PMO 250.273. (B) Articulated fish tail, PMO 250.279. (C) Possible neural spine measuring 4,7 mm in length, PMO 250.267. (D) Fish scale with enamel and growth lines, PMO 250.004. [file peerj-14-20746-s005.png]
